# Supplementary material for: Two opposite voltage-dependent currents control the unusual early development pattern of embryonic Renshaw cell electrical activity
Source: eLife. 2021 Apr 26;10:e62639. doi: 10.7554/eLife.62639 (PMC8139835; doi:10.7554/eLife.62639)
Supplement: Figure 3—source data 1. [file elife-62639-fig3-data1.docx]

| **ID** | **log mean half- amplitude event duration (mean ½Ad)** | **coefficient of variation**  **of ½ Ad ( %)** | **log duration**  **ratio (ddr)** | **cluster membership (k=5)** | **silhouette**  **width** |
| --- | --- | --- | --- | --- | --- |
| 1 | 0,86332 | 0 | -2,4377 | 1 | 0,8474 |
| 2 | 0,86034 | 0 | -2,4407 | 1 | 0,8470 |
| 3 | 0,80018 | 0 | -2,5008 | 1 | 0,8319 |
| 4 | 0,75548 | 0 | -2,5456 | 1 | 0,8117 |
| 5 | 1,0591 | 0 | -1,9409 | 1 | 0,5958 |
| 6 | 0,79389 | 0 | -2,5071 | 1 | 0,8294 |
| 7 | 0,88479 | 0 | -2,4162 | 1 | 0,8477 |
| 8 | 0,82454 | 0 | -2,4765 | 1 | 0,8392 |
| 9 | 0,78533 | 0 | -2,5157 | 1 | 0,8257 |
| 10 | 0,66276 | 0 | -2,6383 | 1 | 0,7621 |
| 11 | 0,6721 | 0 | -2,6289 | 1 | 0,7674 |
| 12 | 0,80346 | 0 | -2,4976 | 1 | 0,8331 |
| 13 | 1,0864 | 0 | -2,2147 | 1 | 0,7798 |
| 14 | 0,76343 | 0 | -2,5376 | 1 | 0,8156 |
| 15 | 0,85126 | 0 | -2,4498 | 1 | 0,8452 |
| 16 | 0,97313 | 0 | -2,3279 | 1 | 0,8344 |
| 17 | 0,74036 | 0 | -2,5607 | 1 | 0,8039 |
| 18 | 0,92942 | 0 | -2,3716 | 1 | 0,8434 |
| 19 | 0,88081 | 0 | -2,4202 | 1 | 0,8478 |
| 20 | 1,281 | 0 | -2,02 | 1 | 0,5816 |
| 21 | 1 | 0 | -2,301 | 1 | 0,8262 |
| 22 | 1,1818 | 0 | -2,1192 | 1 | 0,6982 |
| 23 | 1,0607 | 0 | -2,2403 | 1 | 0,7964 |
| 24 | 1,0607 | 0 | -2,2403 | 1 | 0,7964 |
| 25 | 0,94448 | 0 | -2,3565 | 1 | 0,8408 |
| 26 | 1,0414 | 0 | -2,2596 | 1 | 0,8068 |
| 27 | 1,143 | 0 | -2,158 | 1 | 0,7356 |
| 28 | 1,0934 | 0 | -2,2076 | 1 | 0,7748 |
| 29 | 0,86923 | 0 | -2,4318 | 1 | 0,8479 |
| 30 | 0,81954 | 0 | -2,4815 | 1 | 0,8379 |
| 31 | 0,86923 | 0 | -2,4318 | 1 | 0,8479 |
| 32 | 0,99564 | 0 | -2,3054 | 1 | 0,8278 |
| 33 | 0,91908 | 0 | -2,382 | 1 | 0,8447 |
| 34 | 0,98677 | 0 | -2,3143 | 1 | 0,8307 |
| 35 | 0,86332 | 0 | -2,4377 | 1 | 0,8474 |
| 36 | 1,3181 | 0 | -2 | 1 | 0,5428 |
| 37 | 1,5966 | 0 | -1,7044 | 2 | -0,0839 |
| 38 | 1,2765 | 0 | -2,0246 | 1 | 0,5876 |
| 39 | 1,6053 | 0 | -1,6957 | 2 | -0,0694 |
| 40 | 1,2355 | 0 | -2,0655 | 1 | 0,6384 |
| 41 | 1,1614 | 0 | -2,1397 | 1 | 0,7187 |
| 42 | 0,80618 | 0 | -2,4948 | 1 | 0,8340 |
| 43 | 0,69897 | 0 | -2,6021 | 1 | 0,7819 |
| 44 | 0,65321 | 0 | -2,6478 | 1 | 0,7565 |
| 45 | 1,3464 | 0 | -1,9547 | 1 | 0,4854 |
| 46 | 1 | 0 | -2,301 | 1 | 0,8262 |
| 47 | 1,1461 | 0 | -2,1549 | 1 | 0,7329 |
| 48 | 1,4997 | 0 | -1,8013 | 2 | -0,2502 |
| 49 | 1,4864 | 64,86 | -0,70072 | 2 | -0,0828 |
| 50 | 1,2716 | 18,45 | -0,85336 | 2 | 0,6976 |
| 51 | 1,0812 | 25,61 | -1,1407 | 2 | 0,6366 |
| 52 | 1,1067 | 10,89 | -1,0804 | 2 | 0,5473 |
| 53 | 1,4144 | 41,64 | -0,80748 | 2 | 0,5626 |
| 54 | 1,0317 | 20,71 | -1,0652 | 2 | 0,6172 |
| 55 | 0,9723 | 19,42 | -0,93079 | 2 | 0,6068 |
| 56 | 1,1567 | 21,45 | -0,99816 | 2 | 0,6850 |
| 57 | 0,93752 | 6,98 | -1,2496 | 2 | 0,3191 |
| 58 | 0,91649 | 14,33 | -1,1293 | 2 | 0,4791 |
| 59 | 1,2081 | 21,52 | -0,79192 | 2 | 0,6979 |
| 60 | 1,2389 | 19,12 | -1,0622 | 2 | 0,6760 |
| 61 | 1,3051 | 29,18 | -0,71716 | 2 | 0,6762 |
| 62 | 0,90173 | 25,63 | -1,2854 | 2 | 0,4662 |
| 63 | 1,288 | 71,52 | -0,93384 | 2 | 0,0201 |
| 64 | 1,1043 | 22,88 | -1,2425 | 2 | 0,5839 |
| 65 | 1,0566 | 32,06 | -0,94337 | 2 | 0,6673 |
| 66 | 1,2928 | 24,86 | -1,1052 | 2 | 0,7021 |
| 67 | 1,2218 | 13,8 | -0,90313 | 2 | 0,6500 |

| 68 | 1,4043 | 31,56 | -1,0516 | 2 | 0,7159 |
| --- | --- | --- | --- | --- | --- |
| 69 | 1,5855 | 35,14 | -0,93746 | 2 | 0,6193 |
| 70 | 1,3666 | 44,45 | -1,0313 | 2 | 0,5887 |
| 71 | 1,612 | 24,66 | -0,57509 | 2 | 0,5335 |
| 72 | 1,6661 | 32,29 | -0,78979 | 2 | 0,5674 |
| 73 | 1,2815 | 22,91 | -1,1164 | 2 | 0,6901 |
| 74 | 1,4038 | 25,78 | -0,89714 | 2 | 0,7289 |
| 75 | 1,3312 | 19,39 | -0,73926 | 2 | 0,6951 |
| 76 | 1,3501 | 15,89 | -0,87176 | 2 | 0,6825 |
| 77 | 1,309 | 19,24 | -0,87806 | 2 | 0,7064 |
| 78 | 1,3725 | 48 | -0,81456 | 2 | 0,4693 |
| 79 | 1,1998 | 18,56 | -1,1014 | 2 | 0,6513 |
| 80 | 1,316 | 20,11 | -0,75462 | 2 | 0,7005 |
| 81 | 1,3208 | 22,63 | -1,1351 | 2 | 0,6842 |
| 82 | 1,4276 | 24,79 | -0,75941 | 2 | 0,6957 |
| 83 | 1,5403 | 19,46 | -0,64671 | 2 | 0,5837 |
| 84 | 1,4196 | 18,34 | -0,92719 | 2 | 0,6952 |
| 85 | 1,8454 | 32,41 | -0,55255 | 2 | 0,3574 |
| 86 | 1,7294 | 25,97 | -0,79347 | 2 | 0,5449 |
| 87 | 1,557 | 21,39 | -0,74403 | 2 | 0,6261 |
| 88 | 1,5443 | 9,6 | -0,75665 | 2 | 0,5373 |
| 89 | 1,2776 | 23,73 | -1,1783 | 2 | 0,6694 |
| 90 | 1,5556 | 22,36 | -0,84241 | 2 | 0,6617 |
| 91 | 1,1092 | 27,89 | -0,89079 | 2 | 0,6920 |
| 92 | 1,0934 | 27,76 | -0,8855 | 2 | 0,6866 |
| 93 | 1,1652 | 22,93 | -0,85692 | 2 | 0,6995 |
| 94 | 1,2297 | 31,39 | -1,2262 | 2 | 0,6545 |
| 95 | 1,1358 | 21,34 | -0,88638 | 2 | 0,6858 |
| 96 | 1,1804 | 28,68 | -1,2754 | 2 | 0,6153 |
| 97 | 1,7506 | 33,6 | -0,77233 | 2 | 0,4974 |
| 98 | 1,4219 | 23,15 | -0,76515 | 2 | 0,6989 |
| 99 | 1,4538 | 33,07 | -0,94419 | 2 | 0,6902 |
| 100 | 1,2995 | 29,15 | -1,1565 | 2 | 0,6926 |
| 101 | 1,7435 | 196,79 | -0,41138 | 3 | 0,6705 |
| 102 | 2,2268 | 77,53 | -0,22913 | 4 | 0,6346 |
| 103 | 1,9466 | 156,3 | -0,27524 | 3 | 0,7062 |
| 104 | 2,3993 | 115,91 | -0,29968 | 4 | 0,2762 |
| 105 | 1,5802 | 125,12 | -0,72085 | 4 | 0,0031 |
| 106 | 2,1388 | 89,79 | -0,3171 | 4 | 0,6750 |
| 107 | 1,9409 | 117,61 | -0,66115 | 4 | 0,2677 |
| 108 | 1,928 | 73,27 | -0,41882 | 4 | 0,5796 |
| 109 | 2,08 | 64,49 | -0,37596 | 4 | 0,5073 |
| 110 | 1,8838 | 166,35 | -0,37581 | 3 | 0,7810 |
| 111 | 2,0434 | 164,14 | -0,21628 | 3 | 0,7623 |
| 113 | 2,0725 | 77,57 | -0,45036 | 4 | 0,6498 |
| 114 | 1,9121 | 65,02 | -0,78688 | 4 | 0,3086 |
| 115 | 2,8227 | 0 | -0,1773 | 5 | 0,8056 |
| 116 | 2,864 | 0 | -0,43703 | 5 | 0,8523 |
| 117 | 3,1298 | 0 | -0,17121 | 5 | 0,8057 |
| 118 | 2,2558 | 0 | -0,74425 | 5 | 0,4122 |
| 119 | 2,4526 | 0 | -0,54737 | 5 | 0,6533 |
| 120 | 2,171 | 69,81 | -0,528 | 4 | 0,5735 |
| 121 | 3,5004 | 0 | 0,19939 | 5 | 0,6402 |
| 122 | 3,0037 | 0 | -0,29737 | 5 | 0,8436 |
| 123 | 2,8645 | 0 | -0,43653 | 5 | 0,8524 |
| 124 | 2,7626 | 0 | -0,23745 | 5 | 0,8059 |
| 125 | 2,8116 | 0 | -0,48945 | 5 | 0,8396 |
| 126 | 3,1278 | 0 | -0,17328 | 5 | 0,8066 |
| 127 | 2,7259 | 0 | -0,57512 | 5 | 0,8016 |
| 128 | 2,5478 | 0 | -0,75326 | 5 | 0,6610 |
| 129 | 2,828 | 0 | -0,47301 | 5 | 0,8446 |
| 130 | 3,1641 | 0 | -0,13697 | 5 | 0,7898 |
| 131 | 2,909 | 0 | -0,39201 | 5 | 0,8552 |
| 132 | 2,975 | 0 | -0,32606 | 5 | 0,8490 |
| 133 | 3,1103 | 0 | -0,19078 | 5 | 0,8133 |
| 134 | 2,721 | 0 | -0,58004 | 5 | 0,7988 |
| 135 | 2,5119 | 0 | -0,78915 | 5 | 0,6232 |
| 136 | 2,5353 | 0 | -0,76574 | 5 | 0,6486 |
| 137 | 2,4579 | 0 | -0,84315 | 5 | 0,5579 |
| 138 | 2,8096 | 0 | -0,49147 | 5 | 0,8389 |
| 139 | 2,7168 | 0 | -0,58419 | 5 | 0,7962 |
| 140 | 2,7536 | 0 | -0,54745 | 5 | 0,8154 |

| 141 | 2,8432 | 0 | -0,4578 | 5 | 0,8483 |
| --- | --- | --- | --- | --- | --- |
| 142 | 2,9175 | 0 | -0,38352 | 5 | 0,8550 |
| 143 | 3,0426 | 0 | -0,25845 | 5 | 0,8337 |
| 144 | 3,017 | 0 | -0,284 | 5 | 0,8405 |
| 145 | 2,9708 | 0 | -0,33022 | 5 | 0,8496 |
| 146 | 2,9133 | 0 | -0,38775 | 5 | 0,8552 |
| 147 | 2,8848 | 0 | -0,41623 | 5 | 0,8543 |
| 148 | 3,1169 | 0 | -0,18409 | 5 | 0,8109 |
| 149 | 3,1031 | 0 | -0,19791 | 5 | 0,8157 |
| 150 | 2,6522 | 0 | -0,64878 | 5 | 0,7508 |
| 151 | 1,43504 | 43,93 | -1,16702 | 2 | 0,5901 |
| 152 | 1,125435 | 30,18 | -1,397444 | 2 | 0,5149 |
| 153 | 1,261801 | 33,06 | -1,261078 | 2 | 0,6417 |
| 154 | 1,185184 | 32,65 | -1,416876 | 2 | 0,5251 |
| 155 | 0,8742529 | 31,37 | -1,949656 | 1 | 0,2102 |
| 156 | 1,269617 | 35,91 | -1,554291 | 2 | 0,4326 |
| 157 | 1,381246 | 38,44 | -1,442663 | 2 | 0,5356 |
| 158 | 1,378335 | 38,35 | -1,445574 | 2 | 0,5337 |
| 159 | 1,244574 | 30,92 | -1,579335 | 2 | 0,3817 |
| 161 | 1,414973 | 38,12 | -1,28394 | 2 | 0,6248 |
| 162 | 1,419625 | 44,05 | -1,404266 | 2 | 0,5417 |
| 163 | 1,480582 | 29,72 | -1,218344 | 2 | 0,6573 |
| 164 | 1,279211 | 73,95 | -1,544678 | 2 | 0,1546 |
| 166 | 1,477989 | 21,4 | -1,220943 | 2 | 0,6249 |
| 167 | 1,571359 | 34,85 | -1,127558 | 2 | 0,6464 |
